# Detection and Characterization of Distinct Alphacoronaviruses in Five Different Bat Species in Denmark

**DOI:** 10.3390/v10090486

**Published:** 2018-09-11

**Authors:** Christina M. Lazov, Mariann Chriél, Hans J. Baagøe, Esben Fjederholt, Yu Deng, Engbert A. Kooi, Graham J. Belsham, Anette Bøtner, Thomas Bruun Rasmussen

**Affiliations:** 1 DTU National Veterinary Institute, Technical University of Denmark, Lindholm, DK-4771 Kalvehave, Denmark; chmari@vet.dtu.dk (C.M.L.); march@vet.dtu.dk (M.C.); grbe@vet.dtu.dk (G.J.B.); aneb@vet.dtu.dk (A.B.); 2 The Natural History Museum of Denmark, University of Copenhagen, 2100 Copenhagen, Denmark; hjbaagoe@snm.ku.dk (H.J.B.); esbenfjederholt@gmail.com (E.F.); 3 School of Animal Science, Xichang College, Xichang 615013, China; dengyu127@163.com; 4 Wageningen Bioveterinary Research, 8221 RA Lelystad, The Netherlands; bart.kooi@wur.nl

**Keywords:** *Vespertilionidae*, coronavirus, nucleotide sequencing, phylogenetic analysis, Europe, host restriction

## Abstract

Bat populations harbour a multitude of viruses; some of these are pathogenic or potentially pathogenic in other animals or humans. Therefore, it is important to monitor the populations and characterize these viruses. In this study, the presence of coronaviruses (CoVs) in different species of Danish bats was investigated using active surveillance at different geographical locations in Denmark. Faecal samples were screened for the presence of CoVs using pan-CoV real-time RT-PCR assays. The amplicons, obtained from five different species of bats, were sequenced. Phylogenetic analysis revealed a species-specific clustering with the samples from *Myotis daubentonii*, showing a close resemblance to coronavirus sequences obtained from the same species of bat in Germany and the United Kingdom. Our results show, for the first time, that multiple, distinct alphacoronaviruses are present in the Danish bat populations.

## 1. Introduction

Coronaviruses (CoVs) are a diverse group of large, single-stranded, positive-sense RNA viruses in the family *Coronaviridae*, order *Nidovirales*. Certain CoVs can cause severe diseases in animals and humans. Most recently, the SARS (severe acute respiratory syndrome) and MERS (Middle East respiratory syndrome) epidemics in humans have sparked a renewed interest in coronaviruses. Nucleotide sequencing and phylogenetic analyses have enabled the discovery of spill-over events and back-tracing of the origin of these viruses from their animal hosts.

Bat populations worldwide are now generally considered to be the natural reservoirs for alpha- and betacoronaviruses, with delta- and gammacoronaviruses having avian reservoirs [1]. However, incomplete information exists about which bat species host specific CoVs and which genera and strains of bat CoVs are circulating in Europe [2].

Seventeen species of bats have been registered as occurring in Denmark [3]. They all belong to the family *Vespertilionidae*, with *Myotis daubentonii* (*M. daubentonii*), *Nyctalus noctula*, *Pipistrellus pygmaeus* (*P. pygmaeus*), *Eptesicus serotinus* (*E. serotinus*), and *Vespertilio murinus* being among the most common and widespread. Important Danish hibernacula for some bat species, of mainly the *Myotis* genus, are the limestone mines of Daugbjerg and Mønsted located in the Western peninsula of Jutland, where each autumn, thousands of bats from a large area of Jutland come to spend the cold season in hibernation [3,4,5,6]. Five species of bats are known to occur in Daugbjerg and Mønsted, with estimated winter populations for 2009 of: *M. daubentonii* (16,200 (±1500) and 10,000 (±1000) individuals, respectively); *Myotis dasycneme* (*M. dasycneme*) (approximately 3400 and 4600 individuals, respectively); and low numbers of *Myotis brandtii* (*M. brandtii*), *Myotis nattereri* (*M. nattereri*), and *Plecotus auritus* (*P. auritus*) [5].

The purpose of this study was to investigate the presence of CoVs in healthy Danish bat populations by: (a) active targeted surveillance of bats associated with the hibernacula at Mønsted; and (b) general surveillance by opportunistic sampling around the country from bats in breeding colonies, day roosts, or caught when flying in the field. In addition, we wanted to relate the different viruses found to the various bat species and to make comparisons to the CoV strains reported from neighbouring countries.

## 2. Materials and Methods

### 2.1. Collection of Samples

Samples were collected from bats in Denmark between 2013 and 2017 (see Table 1). The limestone mines of Mønsted were chosen as sampling locations due to their important status as hibernacula. In this study, samples were actively collected at least once a year when the bats departed from the mines during spring or entered during autumn.

At Mønsted limestone mines, bats were captured using a modified version of a harp trap placed in front of the entrance to the mines [7,8,9]. Faecal pellets were collected from individual bats using sterile tweezers. Bats were handled carefully and for the shortest possible time. Bat species were identified based on morphological features. Capturing was performed with permission from the Nature Agency of the Ministry of Environment and Food in Denmark.

**Figure 1:**
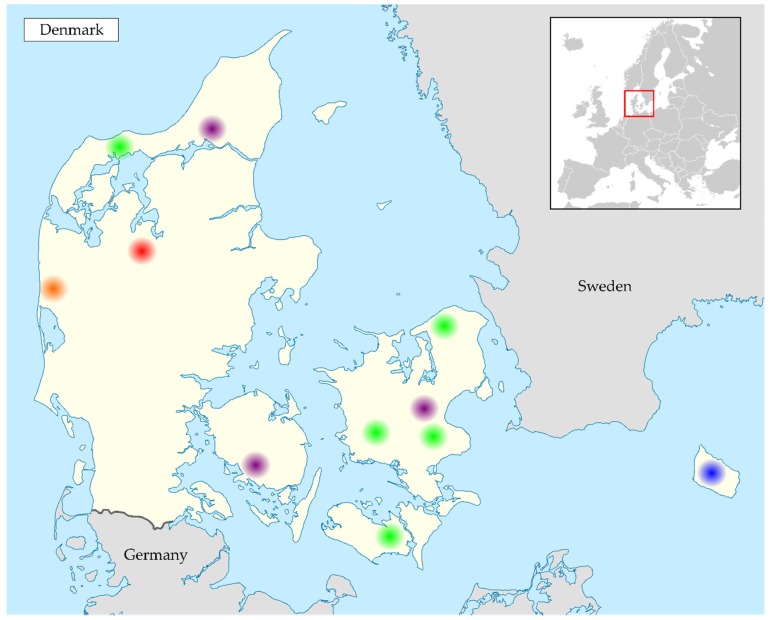
Locations of sampling in Denmark and the sampled bat species. The number of samples collected from each bat species is listed with the number of positive samples shown in parenthesis. (Red) Mønsted, sampled species: *Myotis daubentonii* 183 (43), *Myotis dasycneme* 19 (5), *Myotis nattereri* 3 (1), *Myotis brandtii* 1 (0). (Purple) Locations with positive soprano pipistrelle samples, sampled species: *Pipistrellus pygmaeus* 23 (6). (Orange) Location with positive serotine bat samples, sampled species: *Eptesicus serotinus* 3 (3). (Blue) Bornholm, only negative samples, sampled species: *Myotis nattereri* 7 (0), *Myotis bechsteinii* 11 (0), *Myotis mystacinus* 2 (0), *Myotis brandtii* 2 (0), *Nyctalus noctula* 4 (0), *Plecotus auritus* 2 (0). (Green) Other locations with only negative samples, sampled species: *Myotis daubentonii* 4 (0), *Myotis nattereri* 1 (0), *Pipistrellus pygmaeus* 5 (0), *Eptesicus serotinus* 1 (0). Graphics edited from Wikimedia user NordNordWest published under the conditions of GNU Free Documentation License Version 1.2 or later.

Additionally, faecal samples were collected from bats during the non-hibernating seasons, from spring to autumn, in connection with other projects where bats had to be handled or roosts/colonies had to be visited. Samples were taken directly from individual bats when handled or from the floor under roosting bats (see Figure 1).

### 2.2. Screening of Faecal Samples Using pan-CoV RT-PCR Assays

The faecal samples were homogenized for 1 min in 500 µL Eagle’s medium (in-house production) using the TissueLyser II (Qiagen, Hilden, Germany) followed by centrifugation at 3000 rpm for 5 min. The RNA was extracted from 200 µL of the supernatant using the MagNaPure 96 robot with the MagNa Pure 96 DNA and Viral Small Volume kit (Roche, Basel, Switzerland) and the Pathogen Universal Protocol. Extracted RNA samples were screened for the presence of CoV RNA using two independent real-time RT-PCRs (screening assays B and C, see Supplementary File 1), each based on previously described pan-CoV primers [10,11] targeting conserved regions of the ORF1b which encodes part of the viral replicase complex.

### 2.3. Sequencing of Samples

Amplicons, generated using screening assays B and C, were sequenced to identify the CoVs. The resulting sequences without primer binding regions were 130 bp for the B assay (corresponding to nucleotide position 14911–15040 of the reference sequence NC_009657) and 208 bp for the C assay (nucleotide position 14251–14458). Selected positive samples from the different species of bats were also tested with a nested pan-CoV RT-PCR [12] and amplicons generated, using this assay A, were sequenced for further phylogenetic comparisons. Sequences obtained from this assay were 381 bp in length (corresponding to reference nucleotide position 14493 to 14873). Sequencing was performed using the BigDye Terminator v. 3.1 Cycle Sequencing Kit on an ABI PRISM 3500 DNA analyser (Applied Biosystems, Foster City, CA, USA).

### 2.4. Sequence Analysis

The Danish bat CoV sequences were assembled and analysed using the CLC Main Workbench and subjected to a BLASTn search to identify the CoV genus. The sequences were aligned with selected reference coronavirus strains approved by ICTV [13], as well as relevant and/or more recent European bat coronavirus sequences found by BLAST searching in the NCBI database. The phylogenies were constructed using the Jukes-Cantor Neighbor-Joining method with 1000 bootstrap replicates and a minimum bootstrap value of 50 for phylogenies based on the A and C assays and a minimum of 20 for the B assay-based phylogeny. Nucleotide (nt) sequences were translated to the predicted amino acid (aa) sequences and compared for synonymity using an alignment. All alignments and phylogenies were made in CLC Main Workbench.

### 2.5. Statistical Analysis

Coronavirus prevalence data was analysed using R [14], investigating the effects of season (spring or autumn), year (2013–2016), and bat gender. Odds-ratio [15] and Fisher’s exact test were performed for each variable of season, year, and gender. A significance level of 0.05 was used in the statistical analysis.

### 2.6. Confirmation of Sampled Host Species

A single *M. dasycneme* faecal sample containing a coronavirus very similar to the coronaviruses from *M. daubentonii* was selected for confirmatory species testing, together with an *M. daubentonii* sample as the control, using a bat species determination protocol. Mouth swabs collected from the same animals were submerged in 500 µL phosphate buffered saline and the nucleic acids extracted from 200 µL on the MagnaPure 96 (Roche, Basel, Switzerland) robot using the same kit and protocol as for the faecal samples. The extracted DNA from the mouth swab samples, as well as from the faecal samples, was tested with two different real-time PCR assays targeting the bat mitochondrial DNA; one assay was specific for the cytochrome B gene and the other for the 16S rRNA gene using previously designed primers ([16] and Supplementary File 1). PCR products were visualized on agarose gels, purified using the GeneJet purification kit, and sequenced as above. Sequencing reads were assembled in CLC Main Workbench and consensus sequences were subjected to BLASTn-search to identify the sampled bat species.

## 3. Results

### 3.1. Samples Positive for Coronavirus RNA

In total, 271 faecal samples were obtained; of these, 187 were from *M. daubentonii*, while *P. pygmaeus* was the second most sampled bat with 28 samples and *M. dasycneme* was third with 19 faecal samples. Faecal samples from five of the 10 sampled bat species tested positive for the presence of CoV RNA (as an indicator of the presence of the virus) in screening assays B and/or C. The positive samples were obtained from *M. daubentonii*, *P. pygmaeus*, *M. dasycneme*, *M. nattereri*, and *E. serotinus* (see Table 1 and Table 2). The three most sampled bat species had an observed prevalence of coronavirus RNA in their faeces of around 20–25% (Table 2). Only one out of eleven samples from *M. nattereri* was CoV positive. Three samples from a maternity colony of *E. serotinus* proved positive in the CoV screening, while a fourth sample, collected at a different location, tested negative.

The bats at Mønsted were sampled twice a year, but only a few bats provided a faecal sample when captured departing from the mines after winter hibernation (11 from *M. daubentonii*). Therefore, the majority of samples were collected during autumn (172 from *M. daubentonii*). Due to the seasonally skewed sample collection, it was not possible to infer a seasonal pattern for the presence of virus from the available data. Odds-ratio testing of the different years revealed significantly higher odds (5:1, 95% CI [2; 17]) for the presence of coronaviruses in the year 2015 compared to 2013, but not in the years 2014 or 2016. Odds-ratio testing of bat gender indicated slightly higher odds of coronavirus presence in females than in males (1.4:1, 95% CI [0.7; 2.9]), but this was not significant.

The samples not originating from Mønsted were collected from individual bats caught in the field, from maternity colonies, or bat roosts with single bats from June through October between the years 2013 and 2017. It is not possible to infer a seasonal, year, or gender specific pattern from these sparse samples, as they were collected from different species and from different geographical locations.

### 3.2. Sequence Analysis and Phylogeny

All generated coronavirus sequences belong to the genus *Alphacoronavirus*, as determined by BLASTn searches and phylogenetic analysis, and they are deposited in the European Nucleic Archive (ENA) database under study accession no. PRJEB28001, PRJEB28437, and PRJEB28438.

**Figure 2:**
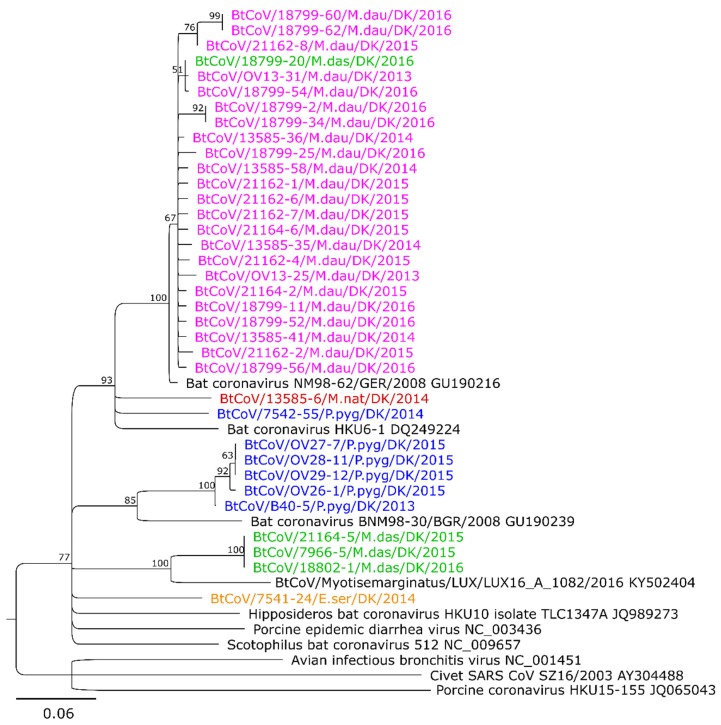
Phylogenetic tree of Danish bat coronavirus sequences. The tree was constructed based on sequenced RT-PCR amplicons from the screening assay C (208 bp without primer sequence [11]). The minimum bootstrap value was set to 50. The Danish CoV sequences are shown in species-specific colours. The distance bar in the bottom left corner indicates the number of nucleotide differences per site.

Separate phylogenetic trees were generated for sequences obtained from the two screening assays C and B (Figure 2 and Supplementary Figure S3). The phylogenetic tree for the 208 bp sequences obtained from screening assay C included 35 Danish CoV sequences (Figure 2). The samples from *M. daubentonii*, *M. dasycneme*, and *M. nattereri* were all collected at Mønsted, while one of the *P. pygmaeus* samples was from an area near Mønsted (Vadum). All the Danish *M. daubentonii* CoV sequences are closely related (>95% nt identity, maximum 9 nt differences), or identical, and form a single cluster within the tree. This cluster also includes one of the Danish *M. dasycneme* CoV sequences and a German CoV sequence from *M. daubentonii* (Accession number GU190216). The latter sequence has only 3 nt differences from the Danish *M. dasycneme* derived sequence and a maximum of 9 nt differences compared to any of the Danish CoV sequences in the cluster. Outside of this cluster, branching from an unresolved node, are two other Danish CoV sequences from *P. pygmaeus* and *M. nattereri*, along with the closest BLAST search result that is the HKU6-1 bat coronavirus from Hong Kong in 2006. This HKU6-1 sequence has at least 24 nt differences compared to the *M. daubentonii* cluster, 25 nt differences to the *P. pygmaeus* sequence, and 29 nt differences to the *M. nattereri* sequence. The five remaining Danish CoV sequences from *P. pygmaeus* form a separate cluster with 0–3 nt differences between them, and with a minimum of 27 nt differences compared to the closest reference virus sequence that was from a sample collected from a *Nyctalus leisleri* specimen in Bulgaria in 2008 (Accession number GU190239). This particular reference sequence was described as forming its own RNA-dependent RNA polymerase-based grouping unit (RGU) in a previous study [17]. The three remaining Danish *M. dasycneme* virus sequences are identical. Their closest reference sequence, with 25 nt differences (out of 208 nt), is from a *Myotis emarginatus* in Luxembourg from 2016. The single Danish CoV sequence from *E. serotinus* does not cluster with any of the other Danish virus sequences, and it branches from an unresolved node.

In the phylogenetic tree based on sequences (120 bp) from the screening assay B, 34 Danish virus sequences were included (Supplementary Figure S3). The *M. daubentonii* sample sequences form a single cluster, which also includes the single *M. dasycneme* sequence. There is a maximum of 5 nt differences within the cluster, but none of the bat CoV reference sequences fall within this cluster. 

The single *P. pygmaeus* and *M. nattereri* virus sequences are similar to, but separate from, this cluster. There are 16 nt differences between these two sequences and from the *P. pygmaeus* to the *M. daubentonii* cluster. The closest reference sequence (KF294382) is from China, with 16 nt differences to the *P. pygmaeus* sequence, 20 nt differences to the *M. nattereri* sequence, and 17 nt differences to the closest *M. daubentonii* viruses.

The other available Danish *P. pygmaeus* CoV sequence is most closely related to a sequence from a *Pipistrellus kuhlii* specimen in Italy from 2010. As in Figure 2, the Danish *E. serotinus* CoV sequence does not cluster with any of the other reference viruses or other Danish CoV sequences, and it branches from an unresolved node. The closest related sequence in the NCBI database is from Kenya (HQ728480), with 20 nt differences.

**Figure 3:**
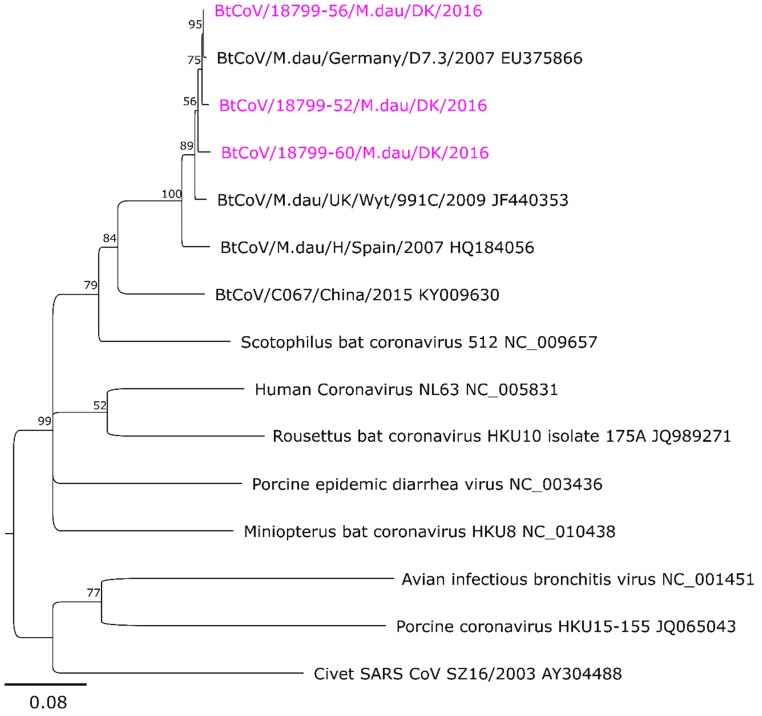
Phylogenetic tree of Danish bat coronavirus sequences. The tree was constructed based on an alignment of 381 bp length references and sequenced RT-PCR amplicons from assay A [12]. The minimum bootstrap value was set to 50. The Danish CoV sequences are shown in pink. The distance bar in the bottom left corner indicates the number of nucleotide differences per site.

Sequences (381 bp in length) were also obtained from selected *M. daubentonii* bat samples using assay A to further characterize the relationships with sequences reported from the same bat species in Germany, Spain, and the United Kingdom [18,19,20]. Samples from other species of bats tested negative in assay A, so no products were available for sequencing. In this tree (Figure 3), the CoV sequences obtained from *M. daubentonii* in Denmark form a cluster with the sequences reported from Germany and from the United Kingdom (UK). There are only 1 to 8 nt differences compared to the German sequence, but 6 to 11 nt differences compared to the U.K. sequences and 17 to 22 nt differences from the Spanish sequence. Between the three Danish samples, there are 4 to 9 nt differences.

### 3.3. Confirmation of Sampled Host Species

The sequenced mouth swab samples yielded a higher quality sequence than the faecal samples in the species identification PCR assays. The sample no. 18799-20 was confirmed to be from the species *M. dasycneme* on the basis of BLASTn searches of the sequenced PCR products from both assays. The PCR products generated with the mitochondrial 16S-gene specific assay were most similar to reference sequences from *M. fimbriatus* and *M. pilosus*, with E-values of 3.55 × 10^−110^ (no 16S reference sequences were found for *M. dasycneme* in GenBank). For the cytochrome B gene-specific assay, the lowest E-value was found for *M. dasycneme* (2.52 × 10^−168^).

The sample 18799-52 was included as the control and was confirmed to be from *M. daubentonii*, with the sequenced PCR products from both assays yielding the lowest BLASTn E-values for *M. daubentonii* references (E-values: 4.14 × 10^−141^ for the 16S PCR product and indistinguishable from 0 for the cytochrome B PCR product).

### 3.4. Predicted Amino Acid Sequences and Host Restriction

**Figure 4:**
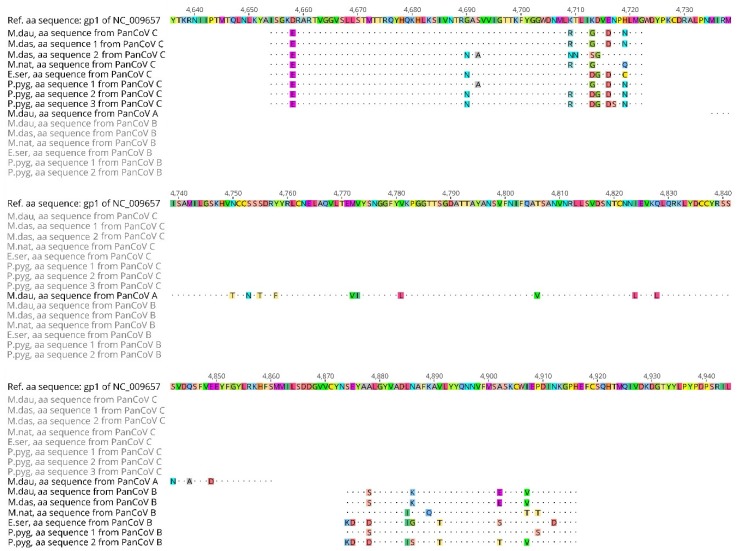
Predicted amino acid sequences derived from the sequenced RT-PCR products of the Danish bat coronaviruses tested with assays A, B, and C. The amino acid sequences are arbitrarily numbered and named according to the sampled bat host species and RT-PCR assay. The most closely related reference sequence Scotophilus bat coronavirus 512 NC_009657 was selected for comparison with the Danish coronavirus predicted amino acid sequences. Numbers refer to amino acid numbers of the annotated gp1 (ORF1ab) of the reference sequence.

All the new nt sequences were translated to their predicted amino acid (aa) sequences and aligned, and identical aa sequences from the same species of bat were removed (see Figure 4).

The predicted aa sequences from all of the *M. daubentonii* samples were identical as the nt sequences within each of the amplicons from each assay only had synonymous nt differences.

For the short B assay amplicons, encoding 43 aa residues between the primer regions, five different aa sequences were found. The sequences had 4–10 aa differences between each other, with the CoVs from *E. serotinus, M. nattereri* and *M. daubentonii* each having one distinct aa sequence and the *P. pygmaeus* CoVs having two. The aa sequence predicted from the single *M. dasycneme* CoV nt sequence was identical to the aa sequence from the *M. daubentonii* samples.

Seven different aa sequences, with 2–8 aa differences between them, were predicted for the C assay amplicon encoding 69 aa between the primer regions. Again, the predicted aa sequence from one of the *M. dasycneme* CoV sequences was identical to the predicted aa sequence from the *M. daubentonii* samples. The other three nt sequences from *M. dasycneme* samples were predicted to encode a single distinct aa sequence. Three different aa sequences were predicted for the six CoV positive samples from *P. pygmaeus*, with four of the samples having identical predicted aa sequences and two nt sequences encoding two distinct aa sequences. The last two different aa sequences were predicted from the two *M. nattereri* and *E. serotinus* coronavirus samples.

## 4. Discussion

In this study, we wanted to investigate the presence of CoVs in healthy Danish bat populations in different geographical locations, including the major colonies at Mønsted, to relate the findings to the bat species and to make comparisons to the bat CoV strains reported from neighbouring countries.

The average prevalence, at around 20%, observed in this study for coronavirus RNA positive faecal samples from the different bat species, is high in comparison with other European studies reporting prevalences of, e.g., 9.8% [18], 4.2% [21], and 1.8% [22]. This could possibly be due to the testing strategy employed, with two different, sensitive screening assays used in this study. Of the 58 CoV positive samples, 48 of these samples provided a nucleotide sequence from at least one of the assays. The remaining 10 samples only yielded partial sequences or were judged positive based on the real-time RT-PCR analysis. Twenty-one of the 48 sequenced samples were positive in both screening assays B and C, 14 samples were only positive in PanCoV C, and 13 only in PanCoV B.

The samples collected from *M. daubentonii* at Mønsted were analysed for seasonal, annual, and gender variability to obtain information about the dynamics of bat coronavirus infection within the bat populations. Previously, reports have been made of a higher coronavirus prevalence in young bats and lactating bats in maternity colonies [18,23,24], suggesting this could be due to the young animals being a susceptible population with a lower immunity that could then amplify the virus and transmit the infection to the adults [18]. Seasonal fluctuations in coronavirus prevalence could be indications of re-introductions of virus and/or the presence of persistently infected animals with periods of virus shedding [25]. Unfortunately, we did not obtain enough samples collected in the springtime to assess seasonal variability in the CoV prevalence in the Danish bats. Our study indicates a higher, but not significant, odds-ratio for females to carry coronaviruses than for males. We observed a significantly higher odds-ratio for coronavirus presence in the *M. daubentonii* samples from Mønsted from the year 2015 compared to years 2013, 2014, and 2016. The reason behind this is unknown.

The co-hibernation of several bat species in one location might facilitate the mixing of viruses between the species. However, the bats at Mønsted are rarely observed to have physical contact with other species, except for occasional aggregations of lethargic hibernating bats. Previously, a high degree of host restriction for the different bat coronavirus strains has been described [2,18,26,27,28]. Therefore, it is relevant to group the detected coronaviruses by bat species and indeed the nt sequencing in this study revealed distinct CoV sequences to be present within each of the five bat species, indicating that specific CoVs are, at least predominantly, restricted to individual host species.

When comparing strains in a phylogeny, it is important to look at variable areas in the genome. In the study by Xu et al., (2003), conserved areas or aa motifs in the ORF1ab encoded RNA-dependent RNA polymerase gene of the SARS coronavirus were inferred by comparison to other coronavirus strains and to other RNA viruses [29]. The Danish bat coronavirus RT-PCR amplicons were generated with primers spanning the nt sequences of some of these motifs. The predicted aa sequence (126 aa) encoded by the region targeted by RT-PCR assay A contains two aa from a conserved motif near its C-terminus, as well as a large motif of 33 aa in the middle of the region. The RT-PCR assay B targets a region encoding 43 aa. This region contains conserved motifs at both ends, as well as an internal motif of 26 aa, meaning that this region of the genome does not represent a variable area for coronaviruses and thus is not optimal for determining phylogeny. The region targeted by RT-PCR assay C encodes 69 aa. The ends of this region likewise contain conserved motifs, but there are no internal conserved motifs. With this in mind, the three different phylogenetic trees should only be regarded as an initial assessment of the geographical connections between the Danish bat coronaviruses and the bat coronaviruses of neighbouring countries. The nt sequences also function as a tool for establishing the genus of the coronaviruses detected in the samples. Furthermore, they provide evidence for species specificity; however, it is not possible to establish firm evolutionary relationships without analysing larger regions of the coronavirus genome that will allow the use of more reference data.

The Danish samples positive for CoV from *M. daubentonii*, *M. dasycneme*, and *M. nattereri* were all collected at Mønsted, but in the phylogenetic trees, there was a clear species-specific clustering, even within the *Myotis* genus. However, a single *M. dasycneme* sample had a coronavirus similar to that found in the *M. daubentonii* bats, and the *M. nattereri* had a distinct, but related, coronavirus to the *M. daubentonii* sequences. The CoV positive samples from *P. pygmaeus* collected from different geographical regions in Denmark, were to some extent, reflected in the genetic relatedness shown in the phylogenetic trees. For instance, one *P. pygmaeus* sequence sampled close to Mønsted grouped close to the Mønsted *M. daubentonii*-cluster. The two other *P. pygmaeus* collection sites, at Borup and Sollerup, are more than 100 km apart and separated by a large stretch of water (Storebælt), but these *P. pygmaeus* coronavirus sequences were still very closely related, with only three synonymous nt differences between the 208 nt sequences in the C assay amplicons from Borup and Sollerup. Unfortunately, the coronavirus present in the positive sample from *E. serotinus* could only be detected using the screening assays and no clear phylogenetic relationship could be established based on the available reference sequences.

The host species differentiation described above became more apparent when the predicted aa sequences derived from the different nt sequences were compared (Figure 4). Here, it was obvious that each species of bat harboured CoVs with distinct predicted amino acid sequences.

From the phylogenetic analysis, we see a close resemblance between the Danish *M. daubentonii* bat CoVs and CoVs from the same bat species in Germany and the U.K., especially in relation to the CoVs from *M. daubentonii* sampled at the Bad Segeberg caves in Germany, located roughly 330 km from the Danish limestone mines [18].

Some bat species in Europe, such as *P. nathusii*, *N. noctula*, and *V. murinus*, are known to be capable of long distance flights. Others, e.g., *M. nattereri*, *Plecotus auritus*, and *E. serotinus,* are rated as much more sedentary, with flights between summer and winter roosts of generally max. 50–100 km. Species like *M. daubentonii* and *M. dasycneme* are normally rated as intermediate, i.e., generally moving or migrating short to medium long distances of up to 300–400 km [30], probably depending on distances to available, suitable, and well-known hibernation or swarming sites [31,32]. However, more recent research indicates that the distinction between these categories is not so sharp [33]. Of the several hundred bats ringed in the Danish limestone mines in the 1950’s and 60’s [34], none have been recaptured in northern Germany and likewise, bats ringed in northern Germany have, to the knowledge of the authors, hardly ever been found in the Danish limestone mines. However, during the intensive population research in Mønsted in 2009 [5], a single *M. dasycneme* female was found that had been ringed as a juvenile the year before in a maternity colony near Kiel in northern Germany. The following year, this individual was captured in northern Germany hibernating in a bunker near Itzehoe, and the following summer, it was back in the colony where it was born (P. Borkenhagen pers. com. cited in [6]).

Furthermore, it has been shown that in late summer and autumn, both *M. daubentonii* and *M. dasycneme* can be frequently observed hunting insects and in more directional flight far at sea in the Baltic [33]. *M. daubentonii* is one of the species that each year gathers at certain points on the coasts of southern Sweden and southern Denmark (Lolland-Falster) and can be seen flying out from these points directly over the sea on what seems to be regular migration. Such *M. daubentonii* specimens in unidirectional flight have been observed in the middle of the Baltic Sea and also migrating out from the south-western point of Bornholm [6,33,35].

Finally, *M. daubentonii* is a very common bat distributed widely and continuously, e.g., in Denmark and northern Germany [4,36], presumably with breeding colonies, intermediate day roosts, etc. all over the region. In the active part of the year, many bats often roost together, in confined spaces in hollow trees, enabling faecal-oral transmission of viruses and it is more than likely that there is frequent contact between animals from neighbouring roosting sites.

In summary, several indications point to a more dynamic situation than previously described, involving actual migration and more flux between *M. daubentonii* and *M. dasycneme* populations in Denmark and northern Germany. This might explain the phylogenetically closely related CoV sequences described in this study. Sequencing of larger and less conserved regions of the coronavirus genome is needed to confirm whether there is, indeed, this close relationship with the bat coronavirus populations of the neighbouring countries.

In this study, only coronaviruses belonging to the genus *Alphacoronavirus* were detected in the Danish bats. These CoVs were obtained from five different species of bats. Four of these species (*M. dasycneme* [18,37], *M. daubentonii* [18,19,20,37], *P. pygmaeus* [18], and *M. nattereri* [20,24]) have previously been reported to harbour coronaviruses of this genus in other countries. To our knowledge, this is the first report of an alphacoronavirus detected from *E. serotinus*. Betacoronaviruses have previously been isolated from both *E. serotinus* [38] and *P. pygmaeus* [39] in other European countries. Therefore, it is likely that Danish bats of these species could also harbour betacoronaviruses, but these were not detected in the limited number of samples analysed in this study. In the five other sampled bat species, no coronavirus positive samples were detected. This could be due to the low number of samples, with only two to nine samples assayed per species. The majority of the samples from these species were collected on the island of Bornholm located in the eastern part of Denmark. Bornholm is the only place where *M. bechsteinii* and *M. mystacinus* were registered during a thorough survey of bat species in Denmark [3]. Of the 24 samples collected from bats in Bornholm, none tested positive for coronavirus RNA; otherwise, CoVs were detected in samples from each of the three major geographical regions of Denmark, namely Jutland, Funen, and Zealand.

## Figures and Tables

**Table 1:** Faecal sample collection details for the detection of coronaviruses in Danish bats.

Bat Species	No. Sampled (No. Positive)	Collection Date	Collection Location	Sample Types
*M. daubentonii*	4 (0)	September 2013	Vesløs	2 individuals and 2 from nest box
	40 (8)	October 2013	Mønsted	Individual
	53 (9)	October 2014	Mønsted	Individual
	4 (1)	April 2015	Mønsted	Individual
	10 (6)	October 2015	Mønsted	Individual
	11 (6)	November 2015	Mønsted	Individual
	5 (0)	May 2016	Mønsted	Individual
	58 (13)	September 2016	Mønsted	Individual
	2 (0)	April 2017	Mønsted	Individual
*M. dasycneme*	2 (0)	October 2013	Mønsted	Individual
	6 (0)	October 2014	Mønsted	Individual
	2 (1)	April 2015	Mønsted	Individual
	1 (1)	November 2015	Mønsted	Individual
	6 (1)	September 2016	Mønsted	Individual
	2 (2)	November 2016	Mønsted	Individual
*M. nattereri*	7 (0)	August–September 2013	Bornholm	Individual
	1 (0)	September 2013	Lolland, Agerup forest	Individual
	2 (1)	October 2014	Mønsted	Individual
	1 (0)	September 2016	Mønsted	Individual
*M. bechsteinii*	4 (0)	August 2013	Bornholm	Individual
	2 (0)	August 2015	Bornholm	1 individual and 1 from nest box
	3 (0)	August 2016	Bornholm	3 nest boxes
	2 (0)	September 2017	Bornholm	Individual
*M. mystacinus*	1 (0)	August 2013	Bornholm	Individual
	1 (0)	August 2016	Bornholm	Individual
*M. brandtii*	2 (0)	August 2013	Bornholm	Individual
	1 (0)	April 2015	Mønsted	Individual
*N. noctula*	4 (0)	August 2013	Bornholm	2 individual samples and 2 from nest boxes
*P. pygmaeus*	6 (1)	August 2013	Funen, Sollerup forest	Individual
	9 (1)	June 2014	Vadum	Mix of individual samples and floor samples from colony
	1 (0)	Summer 2015	Sorø	Sample from nest box
	8 (4)	September 2015	Borup	5 individual samples, 3 from floor
	1 (0)	September–October 2015	Helsinge	Sample from abandoned colony
	3 (0)	August 2016	Tureby	From colony
*P. auritus*	1 (0)	August 2013	Bornholm	Individual
	1 (0)	September–October 2015	Bornholm	Sample from floor
*E. serotinus*	3 (3)	June 2014	Tim	1 individual sample, 2 from floor
	1 (0)	June 2017	Sakskøbing	Sample from floor

**Table 2:** Coronavirus prevalence in the samples from different bat species in Denmark.

Bat Species	Total No. Sampled (No. Positive)	Observed Coronavirus Prevalence in Samples	95% Confidence Interval of Sample Mean
*M. daubentonii*	187 (43)	23%	[0.17; 0.29]
*M. dasycneme*	19 (5)	26%	[0.07; 0.46]
*M. nattereri*	11 (1)	9%	[0.00; 0.26]
*M. bechsteinii*	11 (0)	0%	NA ^1^
*M. mystacinus*	2 (0)	0%	NA
*M. brandtii*	3 (0)	0%	NA
*N. noctula*	4 (0)	0%	NA
*P. pygmaeus*	28 (6)	21%	[0.06; 0.37]
*P. auritus*	2 (0)	0%	NA
*E. serotinus*	4 (3)	75%	[0.33; 1.00]
Total (all species)	271 (58)	21%	[0.17; 0.26]

^1^ NA: not applicable.

## Supplementary Materials

The following are available online at http://www.mdpi.com/1999-4915/10/9/486/s1.
