# Supplementary material for: Detection and Characterization of Distinct Alphacoronaviruses in Five Different Bat Species in Denmark
Source: Viruses. 2018 Sep 11;10(9):486. doi: 10.3390/v10090486 (PMC6163574; doi:10.3390/v10090486)
Supplement: Supplementary file 1 [file viruses-10-00486-s001.zip › Supplementary file 1.docx]

Supplementary file 1

List of protocols for real-time RT-PCR and PCR assays used in this study. Degenerate bases in the primer sequences are indicated with IUPAC standard ambiguity symbols.

PanCoV real-time RT-PCR assay A

1^st^ round real-time RT-PCR

RT-PCR primer sequences as described by [1]:

PC2S2a (5’) TTATGGGTTGGGATTATC (3’)

PC2S2b (5’) TGATGGGATGGGACTATC (3’)

PC2As1a (5’) TCATCACTCAGAATCATCA (3’)

PC2As1b (5’) TCATCAGAAAGAATCATCA (3’)

PC2As1c (5’) TCGTCGGACAAGATCATCA (3’)

1st round primer mix:

PC2S2a and PC2S2b 1 pmol/µl of each

PC2As1a, PC2As1b and PC2As1c 3 pmol/µl of each

Content of one PCR tube of total volume 12.5 μl:

5x One-Step RT-PCR Buffer (Qiagen) 2.5 μl

PanCoV A 1st round primer mix 1.25 μl

dNTP mix (10mM of each) (Qiagen) 0.5 μl

One-Step RT-PCR Enzyme Mix (Qiagen) 0.5 μl

RNase free water 5.25 μl

RNA template 2.5 μl

Cycling temperature program:

50°C 30 min

95°C 15 min

10 cycles of:

94°C 20 sec

62°C 30 sec (with -1°C reduction/cycle)

72°C 40 sec

30 cycles of:

95°C 20 sec

52°C 30 sec

72°C 40 sec

2^nd^ round nested real-time PCR

PCR primer sequences as described by [1]:

PCSa (5’) CTTATGGGTTGGGATTATCCTAAGTGTGA (3’)

PCSb (5’) CTTATGGGTTGGGATTATCCCAAATGTGA (3’)

PCNAs (5’) CACACAACACCTTCATCAGATAGAATCATCA (3’)

2^nd^ round primer mix:

PCSa and PCSb 0.4 pmol/µl of each

PCNAs 4 pmol/µl

Content of one PCR tube of total volume 25 μl:

10x AccuPrime PCR Buffer 1 (Invitrogen) 2.5 μl

PanCoV A 2^nd^ round primer mix 2.5 μl

AccuPrime™ Taq High Fidelity (Invitrogen) 0.1 μl

ResoLight (Roche) 1 μl

RNase free water 17.9 μl

1^st^ round RT-PCR product 1 μl

Cycling temperature program, including melting curve analysis:

95°C 2 min

30 cycles of:

94°C 20 sec

60°C 30 sec (Fluorescence measurement)

72°C 30 sec

1 cycle of:

95°C 1 min

55°C 30 sec (Fluorescence measurements from 55°C to 95°C)

95°C 30 sec

PanCoV real-time RT-PCR assay B

RT-PCR primer sequences as described by [2] :

11-FW (5’) TGATGATGSNGTTGTNTGYTAYAA (3’)

13-RV (5’) GCATWGTRTGYTGNGARCARAATTC (3’)

Content of one PCR tube of total volume 12.5 μl:

5x One-Step RT-PCR Buffer (Qiagen) 2.5 μl

PanCoV B primer mix (7 pmol/µl of each primer) 1.25 μl

dNTP mix (10mM of each) (Qiagen) 0.5 μl

One-Step RT-PCR Enzyme Mix (Qiagen) 0.5 μl

ResoLight (Roche) 0.5 μl

RNase free water 4.75 μl

RNA template 2.5 μl

Cycling temperature program, including melting curve analysis:

50°C 30 min

95°C 15 min

5 cycles of:

94°C 1 min

40°C 1 min

72°C 1 min

30 cycles of:

95°C 1 min

50°C 1 min (Fluorescence measurement)

72°C 1 min

1 cycle of:

95°C 1 min

55°C 30 sec (Fluorescence measurements from 55°C to 95°C)

95°C 30 sec

PanCoV real-time RT-PCR assay C

RT-PCR primer sequences as described by [3]:

Cor-FW (5’) ACWCARHTVAAYYTNAARTAYGC (3’)

Cor-RV (5’) TCRCAYTTDGGRTARTCCCA (3’)

Content of one PCR tube of total volume 12.5 μl:

5x One-Step RT-PCR Buffer (Qiagen) 2.5 μl

PanCoV C primer mix (40 pmol/µl of each primer) 1.25 μl

dNTP mix (10mM of each) (Qiagen) 0.5 μl

One-Step RT-PCR Enzyme Mix (Qiagen) 0.5 μl

ResoLight (Roche) 0.5 μl

RNase free water 4.75 μl

RNA template 2.5 μl

Cycling temperature program, including melting curve analysis:

50°C 30 min

95°C 15 min

50 cycles of:

94°C 30 sec

48°C 30 sec (Fluorescence measurement)

72°C 1 min

1 cycle of:

95°C 1 min

55°C 30 sec (Fluorescence measurements from 55°C to 95°C)

95°C 30 sec

Bat species identification PCR assay for mitochondrial 16S rRNA gene

PCR primer sequences:

Bat-16S-for1 (5’) GACGAGAAGACCCTATGGAG (3’)

Bat-16S-rev1 (5’) AACATCGAGGTCGTAAACC (3’)

Content of one PCR tube of total volume 12.5 μl:

5x One-Step RT-PCR Buffer 2.5 μl

16S primer mix (7 pmol/μl of each primer) 1.25 μl

dNTP mix (10mM of each) (Qiagen) 0.5 μl

One-Step RT-PCR Enzyme Mix 0.5 μl

ResoLight (Roche) 0.5 μl

RNase free water 4.75 μl

RNA template 2.5 μl

Cycling temperature program, including melting curve analysis:

50°C 30 min

95°C 15 min

40 cycles of:

94°C 1 min

45°C 30 sec (Fluorescence measurement)

72°C 1 min

1 cycle of:

95°C 1 min

55°C 30 sec (Fluorescence measurements from 55°C to 95°C)

95°C 30 sec

Bat species identification PCR assay for mitochondrial cytB gene

PCR primer sequences as described by [4]:

CytB Uni fw (5’) TCATCMTGATGAAAYTTYGG (3’)

CytB Uni rev (5’) ACTGGYTGDCCBCCRATTCA (3’)

Content of one PCR tube of total volume 12.5 μl:

5x One-Step RT-PCR Buffer (Qiagen) 2.5 μl

CytB primer mix (7 pmol/µl of each primer) 1.25 μl

dNTP mix (10mM of each) (Qiagen) 0.5 μl

One-Step RT-PCR Enzyme Mix (Qiagen) 0.5 μl

ResoLight (Roche) 0.5 μl

RNase free water 4.75 μl

RNA template 2.5 μl

Cycling temperature program, including melting curve analysis:

50°C 30 min

95°C 15 min

40 cycles of:

94°C 1 min

45°C 30 sec (Fluorescence measurement)

72°C 1 min

1 cycle of:

95°C 1 min

55°C 30 sec (Fluorescence measurements from 55°C to 95°C)

95°C 30 sec

References

1. de Souza Luna, L. K.; Heiser, V.; Regamey, N.; Panning, M.; Drexler, J. F.; Mulangu, S.; Poon, L.; Baumgarte, S.; Haijema, B. J.; Kaiser, L.; Drosten, C. Generic Detection of Coronaviruses and Differentiation at the Prototype Strain Level by Reverse Transcription-PCR and Nonfluorescent Low-Density Microarray. *J. Clin. Microbiol.* **2007**, *45*, 1049–1052, doi:10.1128/JCM.02426-06.

2. Escutenaire, S.; Mohamed, N.; Isaksson, M.; Thorén, P.; Klingeborn, B.; Belák, S.; Berg, M.; Blomberg, J. SYBR Green Real-Time Reverse Transcription-Polymerase Chain Reaction Assay for the Generic Detection of Coronaviruses. *Arch. Virol.* **2007**, *152*, 41–58, doi:10.1007/s00705-006-0840-x.

3. Vijgen, L.; Moës, E.; Keyaerts, E.; Li, S.; Van Ranst, M. A Pancoronavirus RT-PCR Assay for Detection of All Known Coronaviruses. *Methods Mol. Biol.* **2008**, *454*, 3–12, doi:10.1007/978-1-59745-181-9.

4. Schlegel, M.; Ali, H. S.; Stieger, N.; Groschup, M. H.; Wolf, R.; Ulrich, R. G. Molecular Identification of Small Mammal Species Using Novel Cytochrome b Gene-Derived Degenerated Primers. *Biochem. Genet.* **2012**, *50*, 440–447, doi:10.1007/s10528-011-9487-8.

© 2018 by the authors. Submitted for possible open access publication under the
terms and conditions of the Creative Commons Attribution (CC BY) license (http://creativecommons.org/licenses/by/4.0/).
