# Supplementary material for: Detection and Characterization of Distinct Alphacoronaviruses in Five Different Bat Species in Denmark
Source: Viruses. 2018 Sep 11;10(9):486. doi: 10.3390/v10090486 (PMC6163574; doi:10.3390/v10090486)
Supplement: Supplementary file 1 [file viruses-10-00486-s001.zip › Supplementary file legends.docx]

Supplementary file legends

Supplementary file 1 (Word document)

List of protocols for real-time RT-PCR and PCR assays used in this study. Degenerate bases in the primer sequences are indicated with IUPAC standard ambiguity symbols.

Supplementary file 2 (Excel document)

Sample information data and accession numbers for all bat faeces samples tested in this study.

Supplementary file 3 (PNG file)

Phylogenetic tree of Danish bat coronavirus sequences. The tree was constructed based on sequenced RT-PCR amplicons from assay B (130 bp without primer sequence [1]). The minimum bootstrap value was set to 20 instead of 50 (see Figure 2) to increase resolution. The Danish CoV sequences are shown in species-specific colours. The distance bar in the left corner indicates the number of nucleotide differences per site.

Supplementary files 4-6 (fasta files)

Alignment files used to generate the three phylogenetic trees.

Supplementary files 7-9 (PNG files)

Visualization of the alignments used to generate the three phylogenetic trees

References

1. Escutenaire, S.; Mohamed, N.; Isaksson, M.; Thorén, P.; Klingeborn, B.; Belák, S.; Berg, M.; Blomberg, J. SYBR Green Real-Time Reverse Transcription-Polymerase Chain Reaction Assay for the Generic Detection of Coronaviruses. *Arch. Virol.* **2007**, *152*, 41–58, doi:10.1007/s00705-006-0840-x.

© 2018 by the authors. Submitted for possible open access publication under the
terms and conditions of the Creative Commons Attribution (CC BY) license (http://creativecommons.org/licenses/by/4.0/).
