# Supplementary figures and images for: Detection and Characterization of Distinct Alphacoronaviruses in Five Different Bat Species in Denmark
Source: Viruses. 2018 Sep 11;10(9):486. doi: 10.3390/v10090486 (PMC6163574; doi:10.3390/v10090486)

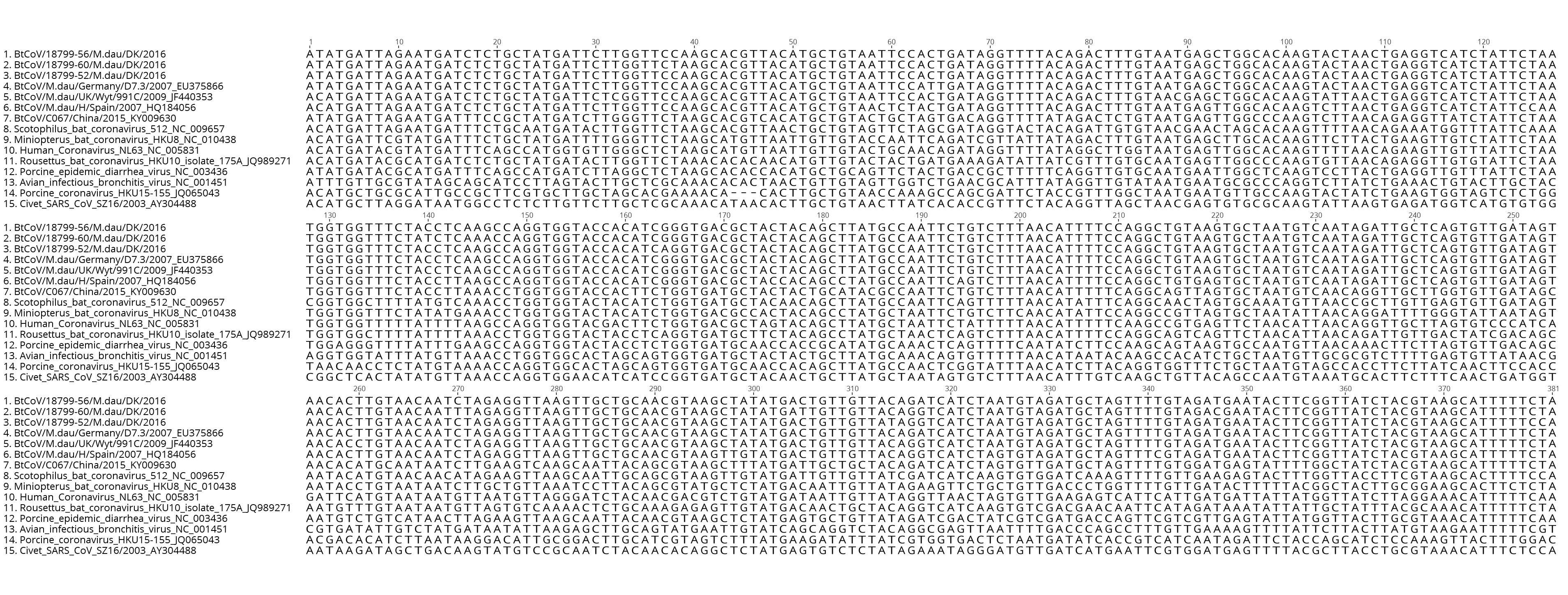

Supplement: Supplementary file 1 [file viruses-10-00486-s001.zip › Supplementary file 7 - PanCoV A alignment.png]

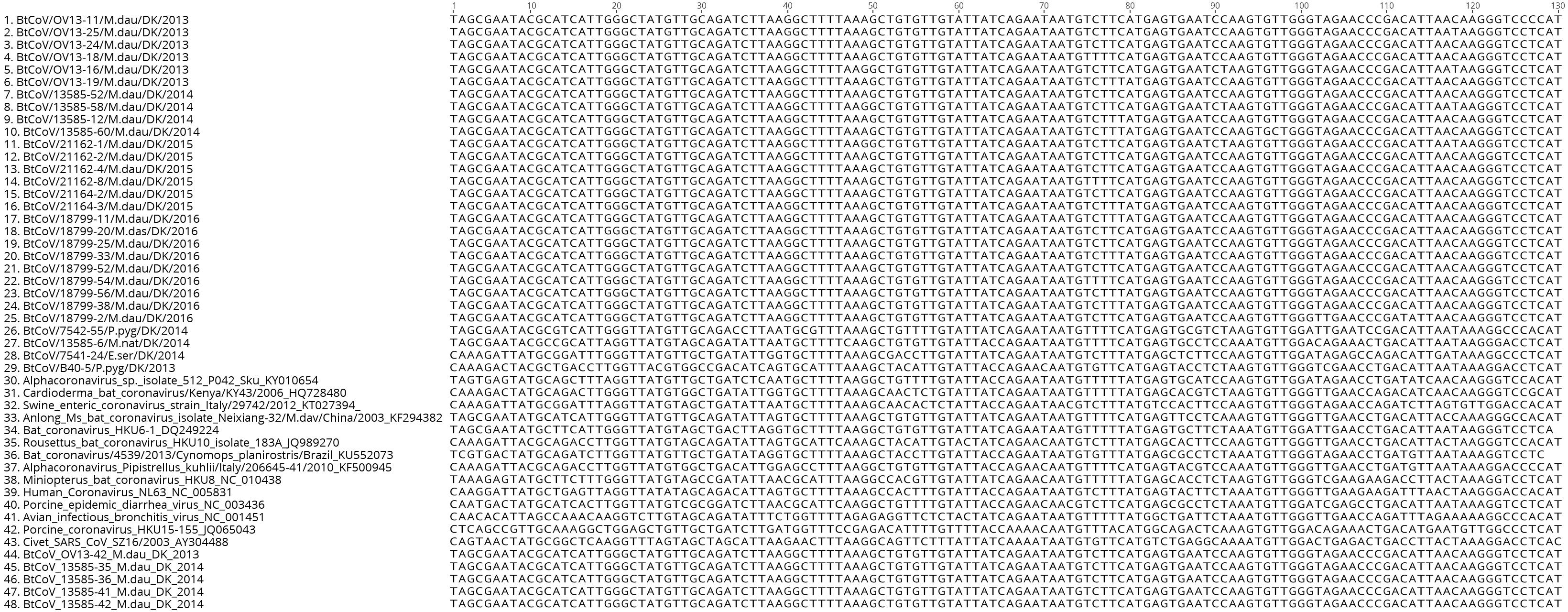

Supplement: Supplementary file 1 [file viruses-10-00486-s001.zip › Supplementary file 8 - PanCoV B alignment.png]

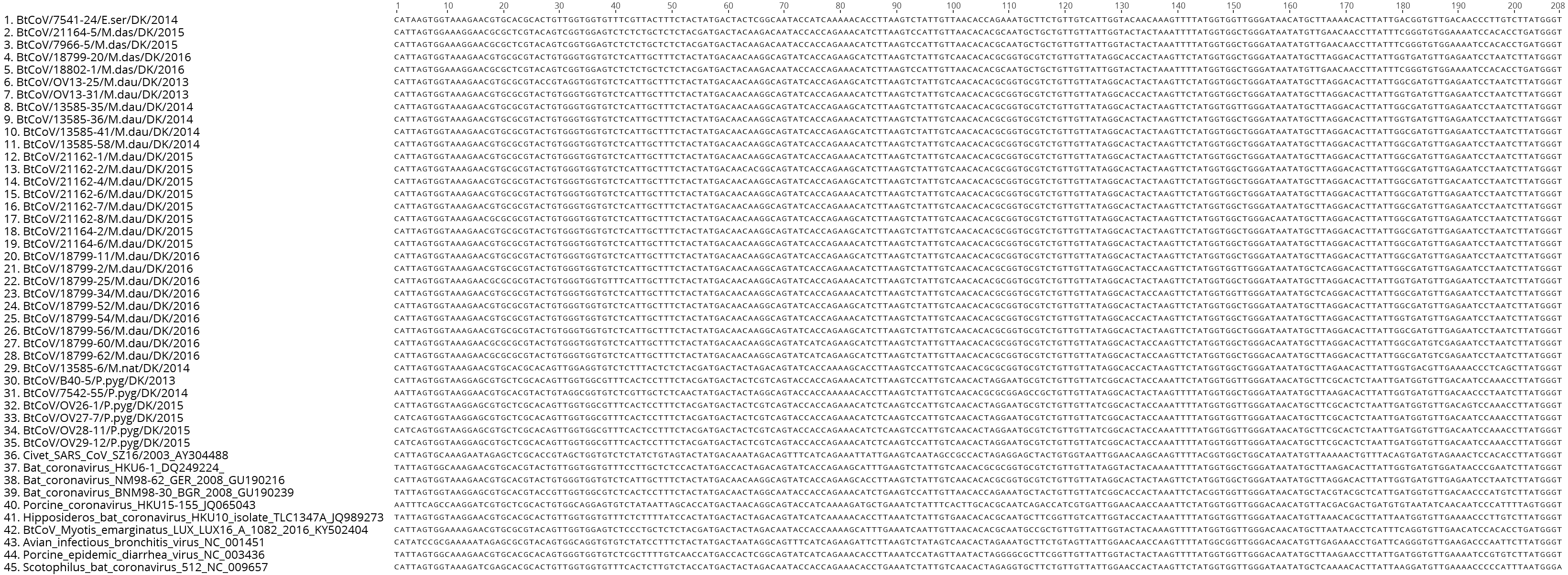

Supplement: Supplementary file 1 [file viruses-10-00486-s001.zip › Supplementary file 9 - PanCoV C alignment.png]
